# Development of anaesthetic protocols for lumpfish (*Cyclopterus lumpus* L.): Effect of anaesthetic concentrations, sea water temperature and body weight

**DOI:** 10.1371/journal.pone.0179344

**Published:** 2017-07-05

**Authors:** Malene W. Skår, Gyri T. Haugland, Mark D. Powell, Heidrun I. Wergeland, Ole B. Samuelsen

**Affiliations:** 1 Department of Biology, University of Bergen, Bergen, Norway; 2 Institute of Marine Research, Bergen, Norway; Swansea University, UNITED KINGDOM

## Abstract

In recent years, use of lumpfish (*Cyclopterus lumpus* L.) as cleaner-fish to remove sea-lice have been chosen by many salmon farmers in Europe and Canada as an alternative to medical treatment, which has led to large scale production of lumpfish. At present, there is limited knowledge of how lumpfish respond upon anaesthesia, which anaesthetics and concentrations that are efficient and conditions for euthanasia. We have therefore tested and developed protocols for bath immersion for three commonly used anaesthetics metacaine (Finquel, buffered tricaine methanesulfonate, MS-222 and Tricaine Pharmaq), benzocaine (Benzoak vet) and isoeugenol (Aqui-S), determined concentration for normal and fast anaesthesia and evaluated safety margin for each condition. Also, a behavioral matrix has been developed. We have examined the effect of fish size (10–20 g, 200–400 g and 600–1300 g) and sea water temperature (6°C and 12°C). We found that 200 mg L^-1^ metacaine is an efficient dose for deep narcosis independently for fish size and temperature due to good safety margins with regards to both exposure times and doses. However, for many tasks lighter anaesthesia is sufficient, and then 100 mg L^-1^ metacaine can be used. Benzocaine is less efficient than metacaine, but can be used as anaesthetic of fish < 400 g. The optimal doses of benzocaine were 100–200 mg L^-1^ for small fish (10–20 g) and 200 mg L^-1^ for medium sized fish (200–400 g). For larger fish (> 600 g), benzocaine is not suitable. Isoeugenol cannot be recommended for full anesthesia of lumpfish. The conditions for lethal doses varied with chosen anaesthetic, fish size and temperature. For small fish (10–20 g), exposure to 1600 mgL^-1^ of metacaine in 10 minutes it lethal. Guided protocols for non-lethal anaesthesia will contribute to ensure safe treatment of lumpfish according to an ethical standard for good fish welfare.

## Introduction

Lumpfish (*Cyclopterus lumpus* L.) have shown to efficiently remove salmon lice (*Lepeophtheirus salmonis* Krøyer) from farmed Atlantic salmon (*Salmo salar* L.) at both temperate and cold temperatures [1,2]. In recent years, lumpfish are therefore chosen by many fish farmers in Norway, and more recently also in Scotland, Ireland, Faroe Islands and Canada, as an alternative to medical treatment. This has led to an increased demand for this cleaner-fish and the number of farmed lumpfish increased in Norway alone from around 0.4 million in 2012 to about 13.3 million in 2015 (http://www.fiskeridir.no/English/Aquaculture/Statistics/Other-marine-fish-species_buying, sale of farmed cleaner fish). In fish farming, each individual`s welfare is an important issue and there are a number of conditions such as transportation, vaccination, blood sampling, egg stripping, culling and various research experiments that may induce stress on the fish and involve potentially painful operations. In such circumstances, the use of anaesthetic agents may be beneficial or even required. Establishment of optimal anaesthetic protocols and useful criteria for monitoring depth of anaesthesia is of particular importance for lumpfish as they often adhere to the substrate by the suction disc rather than swim actively, and it may be difficult to evaluate if they are anaesthetized or not.

The response of fish to stressful conditions consists of a wide range of external and internal alterations comparable to the physiological stress response displayed by higher vertebrates [3, 4]. A variety of stimuli can induce stress and while pain and fear are among the strongest stressors in higher vertebrates, there are still opposing views among researchers whether teleost fish are able to experience fear or have the mental capacity for awareness of pain and suffering [5–9]. However, studies on rainbow trout (*Oncorhynchus mykiss* Walbaum) and goldfish (*Carassius auratus* L.) have demonstrated that fish possess the basic neural system necessary for nociception, i.e. perception of painful stimuli [5,8–11]. Anaesthetics in commercial fish farming are mainly used for immobilisation of fish in order to facilitate handling, but are also applied in other situations ranging from mild sedation during transport to full anaesthesia during more invasive procedures. The most common route of administration of anaesthetics to fish is dispersion in the water and absorption across the gills. The effect is usually assessed by induction and recovery time, reflex reactions to external stimuli and responsiveness to handling.

Among the most frequent used anaesthetics in aquaculture worldwide are metacaine (Finquel, MS-222, Tricaine Pharmaq, TMS, tricaine methanesulfonate, ethyl 3-aminobenzoate), benzocaine (ethyl 4-aminobenzoate), quinaldine, 2-phenoxyethanol, metomidate and isoeugenol (2-methoxy-4-prop-1-enyl-phenol) [12–16]. The three most used anaesthetics in Norwegian aquaculture in recent years are, according to the statistic, benzocaine, metacaine and isoeugenol (www.fhi.no). To ensure the welfare of the fish subjected to procedures that might inflict pain, anaesthetic agents with the ability to block nociceptive pathways are necessary. The three anaesthetics benzocaine, metacaine and isoeugenol possess this ability. When applied in human and veterinary medicine, metacaine and benzocaine are local anaesthetics that act by blocking voltage-sensitive sodium channels [17–18], thereby preventing the voltage-dependent increase in sodium conductance. This inhibits the initiation and propagation of action potentials in excitable cells; thus blocking most neurons and muscle cells and are therefore causing paralysis in addition to blocking nociception. When administrated to fish via bath immersion, they enter the circulation and produce general anaesthesia by inhibiting neural signal transmission ranging from the periphery to higher parts of the nervous system. The precise mechanism of action in the central nervous system is however not fully understood [19–21]. A high dose of metacaine and benzocaine can be used for euthanasia [22]. Isoeugenol is structurally similar to eugenol, a widely used analgesic in dentistry that inhibits sodium, potassium and calcium channels, inhibits NMDA receptors and potentiates GABA_A_ receptors [23–26]. Good anaesthetic effect, rapid induction and recovery times and good safety margins are important properties for fish anaesthetics [27–28]. Dosage regimes maintaining these properties vary between agents and between fish species and are also influenced by biological factors such as body weight, age and sex, as well as environmental factors such as salinity, pH, oxygen level and water temperature [16, 29–31]. Therefore it is essential to develop optimal protocols for each species. Also, lethal doses should be determined for each species as anaesthetic overdose is a commonly used method to kill fish [22].

In the currently study we have developed anaesthetic protocols for lumpfish of various sizes at two different temperatures, and investigated the safety margins under each conditions by determining the concentrations of the agents and exposure times that give anaesthetic overdose.

## Materials and methods

The described experiment was approved by the Norwegian Animal Research Authority (Identification number 8234). Fish were sacrificed with overdose of anaesthetics or by a sharp blow to the head, which is an appropriate procedure under Norwegian law.

### Fish

Farmed lumpfish (*C*. *lumpus* L.) were supplied by Fjord Forsk Sogn AS, a commercial breeder in Sogn & Fjordane County, Norway. The fish were transported by road using a commercial fish transporter vehicle, which maintained stock at satisfactory temperature and dissolved oxygen concentration. The fish were kept in fish tanks (500 L) at the Aquatic and Industrial Laboratory (ILAB), Bergen, Norway, until they had reached the preferred size. Two water temperatures were applied (6 and 12°C) and the light regime 12 hours light: 12 hours dark. The temperatures were selected to mimic winter (6°C) and summer (12°C) temperatures. This temperature range also cover temperatures in most production sites. The size of the fish tanks were 500 L and the outlet water had a minimum of 77% oxygen saturation. The water flow, 34 PSU, was 300–400 L per h per tank. The fish were fed (3% of body weight) with the commercial dry feed Amber Neptune (Skretting AS, Norway), a marine feed developed for gadoids.

The fish were divided into three groups according to their size (independent of sex) and included small fish (10–20 g), medium sized fish (200–400 g) and large fish (600–1300 g). The experiments were conducted at ILAB.

### Anaesthetics

**Fig 1:**
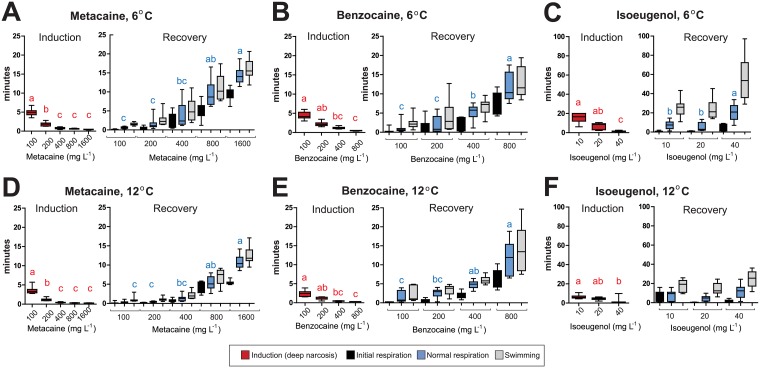
Induction and recovery times during immersion anaesthesia with metacaine, benzocaine and isoeugenol of small fish (10–20 g). Induction and recovery with different doses of (A) metacaine at 6°C, (B) benzocaine at 6°C, (C) isoeugenol at 6°C, (D) metacaine at 12°C, (E) benzocaine at 12°C, (F) isoeugenol at 12°C. The colored boxes show times for induction and recovery. Average time in minutes ± S.D. is shown. Red boxes = induction times (no respiration), black boxes = initial respiration, blue boxes = normal respiration, grey boxes = swimming activity. Single letters means that this measurement is statistically different the other. Shared letters means that the differences are not statistically significant (P < 0.05). N = 10 for all anaesthetic concentrations, except for isoeugenol at 6°C for which N = 5. Statistical analyses for induction and each of the phases during recovery, as well as statistical analyses of the effect of temperature, are given in S4–S7 Tables.

**Fig 2:**
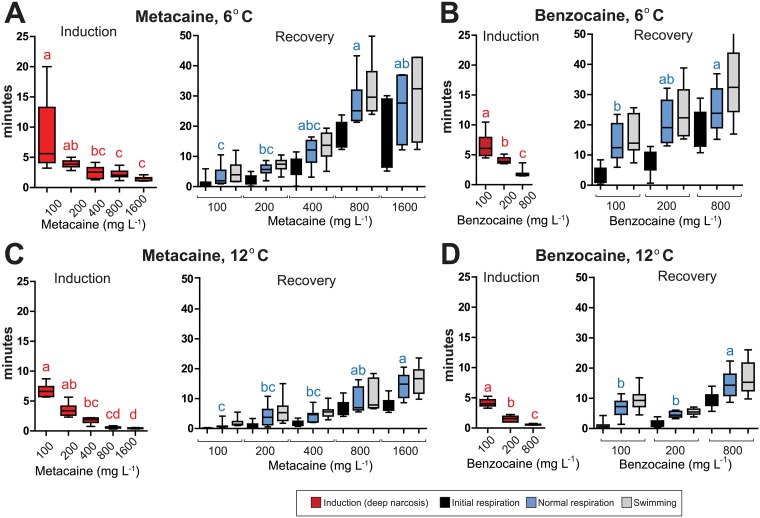
Induction and recovery times during immersion anaesthesia with metacaine and benzocaine of medium sized fish (200–400 g). Times for induction and recovery with different doses of (A) metacaine at 6°C, (B) benzocaine at 6°C, (C) metacaine at 12°C, (D) benzocaine at 12°C. The colored boxes show times for induction and recovery. Average time in minutes ± S.D. is shown. Red boxes = induction times, black boxes = initial respiration, blue boxes = normal respiration, grey boxes = swimming activity. Single letters means that this measurement is statistically different the other. Shared letters means that the differences are not statistically significant (P < 0.05). For all anaesthetic concentrations N = 10, except for metacaine 1600 mg L^-1^ at 6°C and benzocaine 800 mg L^-1^ 6°C for which N = 5. Statistical analyses for induction and each of the phases during recovery, as well as statistical analyses of the effect of temperature, are given in S4–S7 Tables.

**Fig 3:**
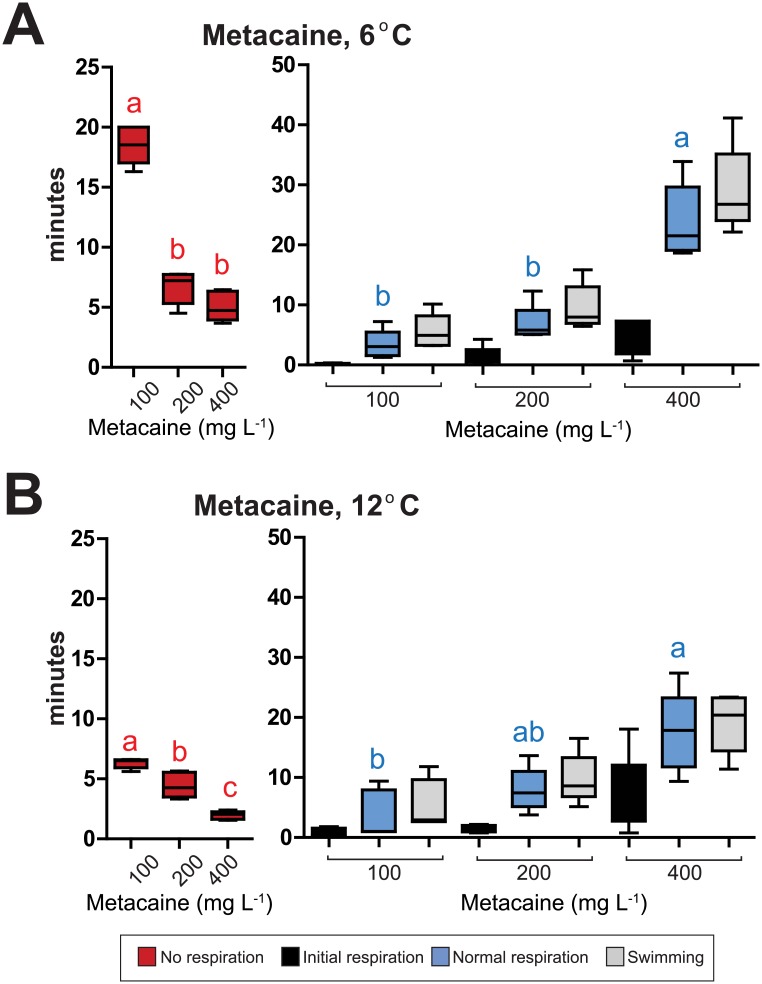
Induction and recovery times during immersion anaesthesia with metacaine of large fish (600–1300 g). Induction and recovery times with different doses of (A) metacaine at 6°C, (B) metacaine at 12°C. The colored boxes show times for induction and recovery. Average time in minutes ± S.D. is shown. Red boxes = induction times, black boxes = initial respiration, blue boxes = normal respiration, grey boxes = swimming activity. Single letters means that this measurement is statistically different the other. Shared letters means that the differences are not statistically significant (P < 0.05). N = 5. Statistical analyses for induction and each of the phases during recovery, as well as statistical analyses of the effect of temperature, are given in S4–S7 Tables.

**Fig 4:**
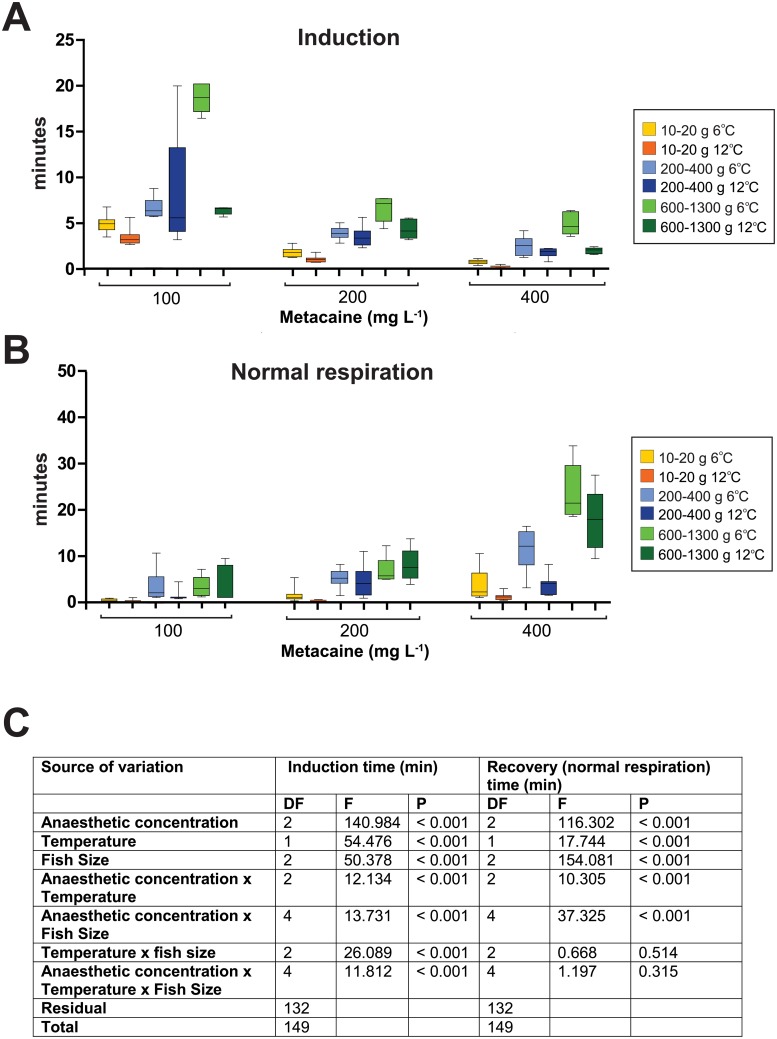
Induction and recovery times during immersion anaesthesia with metacaine. (A) Times for induction for all fish sizes at two temperatures for three different doses of metacaine. (B) Times for recovery (normal respiration). The boxes show average with standard deviation. Yellow boxes = fish 10–20 g at 6°C, orange boxes = fish 10–20 g at 12°C, light blue boxes = fish 200–400 g at 6°C, dark blue boxes = fish 200–400 g at 12°C, light green boxes = fish 600–1300 g at 6°C, dark green boxes = fish 600–1300 g at 12°C. (C) Statistical analyses of effect of Anaesthetic concentrations, temperature and fish size alone or in combination.

The anaesthetics included in this study were metacaine (Finquel, also known as MS-222, TMS, tricaine methanesulfonate, Tricaine Pharmaq), benzocaine and isoeugenol. To prepare the doses of metacaine, powder of Finquel (Scan Aqua AS, Årnes, Norway) was added directly to the anaesthetic chamber containing sea water. The concentrations tested were 100, 200, 400, 800 and 1600 mg L^-1^. To prepare the doses of isoeugenol, Aqui-S vet (Scan Aqua AS, Årnes, Norway) containing 540 mg mL^-1^ of isoeugenol was diluted 1:10 in milliQ water prior to be added to the anaesthetic chamber containing sea water. The concentrations tested were 10, 20 and 40 mg L^-1^. A stock solution of benzocaine was made by dissolving 200 g (powder) in 1 L ethanol. From the stock solution appropriate volumes were added to the anaesthetic chamber to obtain concentrations of 100, 200, 400 and 800 mg L^-1^. The anaesthetic concentrations used for the different groups of fish, shown in Figs 1–4, were determined based on preliminary tests applying two to five fish for each dose.

### Immersion anaesthesia with metacaine, benzocaine and isoeugenol

Anaesthetic solutions for immersion anaesthesia were prepared as described in section 2.2. The sea water used in the experiments was obtained from the fish tank. Oxygen level and temperature were monitored during the procedure. The fish were anaesthetized one by one (N = 10 for small and medium sized fish and N = 5 for the largest fish) and the anaesthetic solutions were replaced after every fifth fish. The chambers for anaesthesia and recovery were opaque, white containers. The volumes of anaesthetic solutions in the tanks were 2 L in 2L containers for small fish (10–20 g) and 10 L in 12 L containers for medium fish (200–400 g) and large fish (600–1300 g). Since lumpfish has a different behavior than other fish species, e.g. salmon, a behavioral matrix tailored to lumpfish was developed. The recorded stages during anaesthesia were disoriented, loss of equilibrium, reduced swimming activity, no swimming activity, near absence of respiration (operculum movements). When the respiration was near absent the fish were transferred to a recovery tank containing sea water with the same temperature as the exposure tank. This is referred to as 0-exposure. The sea water in the recovery tank was replaced after every 3–5 fish. The recorded stages in the recovery phase were initial and strong movement of operculum, initial movement of fins, starting point for swimming, normal swimming activity and when equilibrium was reestablished. After the recovery phase the fish were transferred to a storage tank and if a fish was anaesthetized more than once, the quarantine was set to 14 days to ensure that the fish was fully recovered. No fish died after 0-exposure. The time to reach each stage during anaesthesia and recovery was recoded for all fish, and time measurements during anaesthesia and recovery for metacaine, benzocaine and isoeugenol are given in S1, S2 and S3 Tables, respectively.

### Determination of therapeutic window for metacaine, benzocaine and isoeugenol

To determine the therapeutic window for each drug at different concentrations, fish sizes and sea water temperatures, the fish were kept in the anaesthetic bath after the respiration were near absence for 10, 30 and 60 min (N = 5) before transfer to a recovery tank. If the fish did not wake up after 1 hour in the recovery tank, they were registered as dead.

### Statistic

Statistical analyses were performed using Sigma Stat v3.5 (Systat Software Inc, Richmond, USA). To evaluate the effect of anaesthetic concentrations within one group (defined by fish size), one way ANOVA with a post hoc Holm-Sidak method or ANOVA on Ranks (Kruskal-Wallis One Way Analysis of Variance on Ranks) with post-hoc Tukey test was performed. To test if there were significant differences between two temperatures, a Mann-Whitney Rank Sum Test was performed for each anaesthetic concentration. Differences were consider significant if P < 0.05. The statistics for induction and normal respiration are shown in the Figs 1–3. A complete summary of statistical analyses for induction, initial respiration, normal respiration and swimming are given in S4, S5, S6 and S7 Tables, respectively. Single letters means that one group is statistically different from the other groups. To determine if there was an interaction between anaesthetic concentrations, temperatures and fish sizes three-way ANOVA were used. Differences were consider significant if P < 0.05.

## Results

### Behavior of lumpfish during anaesthesia and response to external stimuli

Lumpfish have different behavior compared to many other fish species and they often use their suction disk to adhere to the substrate rather than swim actively. Therefore, an important first-step in the evaluation of efficient anaesthetic was to determine the behavior correlating with different stages of anaesthesia for lumpfish. The physical signs during the different stages were disorientation, loss of equilibrium, reduced swimming activity, no swimming activity, cessation of fin movements and no respiration (cessation of operculum movement) (Table 1). Also, erratic behavior with increased respiration and swimming activity was observed during anaesthesia with high concentrations (> 400 mg L^-1^) of metacaine and occasionally at high concentrations of benzocaine. For lumpfish, this excitatory stage occurred prior to the sedation stage.

**Table 1:** Overview of behaviour of lumpfish at different stages of anaesthesia ^1^.

Stage	Plane	Description	Appearance	Swimming activity	Equilibrium	Responsiveness^2^	Muscle tone	Respiration
**0**		Normal	Normal	Normal/adhere to the substrate	Normal	Yes	Normal	Normal
**I**		Light sedation	Disoriented	Reduced	Normal/ reduced	Reduced	Normal	Normal
II*		Excitatory	Excited	Increased	Struggles to maintain balance	n.d.	Normal	Irregular/ increased
**III**	1	Light anaesthesia	Anaesthetized	Stopped	Lost	None	Decreased	Normal or decreased
	2	Surgical anaesthesia	Anaesthetized	Stopped	Lost	None	Relax	Shallow
	3	Deep narcosis	Anaesthetized	Stopped	Lost	None	None	Nearly absent
**IV**		Impending death	Moribund	Stopped	Lost	None	None	Stopped

^1^ The Table has been modified from Zahl et al. [32]

^2^ Responsiveness refers to reaction to external stimuli (pinch in the lower lip)

*Observed at high concentrations of metacaine (> 400 mg L^-1^)

Upon exposure to benzocaine, medium and large fish swam laterally for a long period (up to several minutes), while during anaesthesia with isoeugenol feces in the water was commonly observed. To evaluate the depth of anaesthesia, the lumpfish’s reaction to external stimuli was monitored. Pinch in the tail and pinprick in the skin where not useful stimuli to provoke a reaction, but we found that pinch in the lower lip induced a bite response. This was the best stimuli to provoke a reaction and was further used this to evaluate if the fish were fully anaesthetized.

### Effect of anaesthetics, anaesthetic concentrations and sea water temperatures on small fish (10–20 g)

For the small fish (10–20 g) metacaine, benzocaine and isoeugenol could all be used as anaesthetics. The induction time to obtain deep narcosis was indirectly proportional to the anaesthetic concentrations independently of temperature, and there were correlations between concentrations of anaesthetics and the recovery times. Higher concentrations resulted in longer recovery times (Fig 1). A summary of time measurements for induction and recovery at different temperatures and concentrations are summarized in S1 Table (metacaine), S2 Table (benzocaine) and S3 Table (isoeugenol), respectively. In the Figures, the time to reach stage III plane 1 (no swimming) is not shown, but this information is given in the Supplementary Tables. Statistical analyses are summarized in S4–S7 Tables. For metacaine at 6°C, there were a statistical significant difference between anaesthetic concentrations and induction times (H_4_ = 43.888 (P < 0.001)) (Fig 1). The same pattern for induction was observed for metacaine at 12°C (H_4_ = 43.028 (P < 0.001)), but the induction times were significantly lower at 12°C compared to 6°C for all doses (S4 Table).

The average recovery times, defined by initial respiration, normal respiration and swimming activity, were less than three minutes for the two lowest concentrations of metacaine (100 and 200 mg L^-1^) at both 6°C and 12°C, and for 400 mg L^-1^ metacaine at 12°C. The time resumed for swimming activity at 400 mg L^-1^ metacaine at 6°C was 5.34 minutes, but there were large differences within the group. There were also large differences at higher concentrations at 6°C (Fig 1A). At increasing concentrations, there were a significant increase in time to reach initial respiration (at 6°C, H_4_ = 43.109 (P < 0.001) and at 12°C, H_4_ = 42.234 (P < 0.001)). The time to reach normal respiration and swimming activity was also significantly higher with increased concentrations of anaesthetics (S6 and S7 Tables, respectively).

The same trends with lower induction times and higher recovery phase with increasing concentrations of anaesthetic were also observed for benzocaine (Fig 1B and 1E) and isoeugenol (Fig 1C and 1F). The induction times for the lowest concentration of benzocaine (100 mg L^-1^) were 4.5 ± 2.0 min at 6°C and 2.4 ± 0.3 min at 12°C (Fig 1B and 1C) and S2 Table. Regarding induction times, there were significant differences between the two temperatures (see S4 Table). Average time to obtain swimming activity were < 5 min for the two lowest concentrations, but increased to about 7 minutes for 400 mg L^-1^ benzocaine and about 12 min for 800 mg L^-1^ (Fig 1B and 1E). For isoeugenol, the average induction time was 14.1 ± 2.2 min at 6°C and 6.5 ± 2.0 min at 12°C for the lowest concentration (10 mg L^-1^). The time to attain full recovery was prolonged for isoeugenol. To reach normal respiration, the average time was 7.33 ± 4.5 min at 6°C and 7.2 ± 4.5 min at 12°C. The induction times at higher concentration decreased, but the time to reach normal respiration increased dramatically. At 40 mg L^-1^, it took 20.7 ± 8.9 min and 12.0 ± 6.9 min before the fish obtained normal respiration, and 55.6 ± 22.2 min (6°C) and 24.5 ± 8.6 min (12°C) before the fish retained swimming activity. There were not significant differences in recovery times between the two temperatures for most of the concentrations of benzocaine and isoeugenol.

### Effect of anaesthetics, anaesthetic concentrations and sea water temperatures on small fish (200–400 g)

Pilot experiments with isoeugenol gave extended induction and recovery times for all concentrations tested and it was therefore concluded that isoeugenol was not a viable alternative for full anaesthesia to the stage of deep narcosis of medium sized lumpfish (200–400 g).

For metacaine and benzocaine the induction time was inverse proportional to anaesthetic concentrations, while the recovery time increased with increased concentrations of anaesthesia. The induction time for the lowest dose (100 mg L^-1^) of metacaine was 8.4 ± 6.4 min at 6°C and 6.7 ± 1.0 min at 12°C (Fig 2A and 2C). Huge variations at 100 mg L^-1^ 6°C are mainly because two of the lumpfish did not reach the deep narcosis stage and were transferred to the recovery tank after 20 minutes. The recovery times for > 400 mg L^-1^ metacaine at 6°C were prolonged and there were also high variation within each group at 6°C, suggesting that these conditions are not recommended.

Upon anaesthesia with benzocaine, the induction time was statistically reduced with increasing concentrations at both temperatures (at 6°C: H_2_ = 24.050 (P < 0.001) and at 12°C: H_2_ = 25.835 (P < 0.001)) (Fig 2B and 2D). Due to long recovery times at 6°C (Fig 2B), benzocaine is not recommended for deep narcosis for medium sized lumpfish. Time for induction and recovery upon anaesthesia with benzocaine were significantly shorter at 12°C compared to 6°C (Fig 2B and 2D, S2 and S4–S7 Tables).

### Effect of anaesthetics, anaesthetic concentrations and sea water temperatures on large fish (600–1300 g)

In pilot experiments, long induction and recovery times were found for both isoeugenol and benzocaine and neither were found suitable as alternatives for full anaesthetic to the stage of deep narcosis for fish of this size. For metacaine, the induction time at a dose of 100 mg L^-1^ at 6°C was too long (18.5 ± 1.5 min) to be considered a viable alternative (Fig 3, S1 Table). Effect of concentrations were statistically significant (F_2,14_ = 138.061 (P < 0.001)). The induction times at concentrations of 200 and 400 mg L^-1^ at 6°C (6.7 ± 1.4 min and 4.5 ± 1.1 min) are within acceptable limits. Upon 400 mg L^-1^ the recovery time was very long (Fig 3A). It took more than 20 min before the fish obtained normal respiration rate and the fish started to swim. Therefore, 200 mg L^-1^ was the only concentrations that gave acceptable induction and recovery times at 6°C.

At 12°C, all three concentrations obtain acceptable induction times (Fig 3B) but for the highest dose, 400 mg L^-1^, the recovery times was very long (21.0 ± 7.7 min). It was a significant effect of temperature on induction times (S4 Table), but not on recovery times (S5–S7 Tables). The dose of 200 mg L^-1^ metacaine is therefore the recommended dose for the large fish at both temperatures. Due to a limited number of fish available of this size only 10 min exposure was used in the safety margin test. No mortality was registered after 10 min exposure at any of the tested concentrations (100–400 mg L^-1^).

### Effect of anaesthetic concentration of metacaine, fish size and temperature

It is beneficial to have a drug that is efficient independently of fish size and temperature and with good safety margin with regard to exposure time and anaesthetic concentrations. Therefore, the inductions and recovery times (normal respiration) between different concentrations of anaesthetic, fish size and temperature were compared (Fig 4) to find the optimal dose. Only metacaine was considered as this was the only anaesthetic suitable as anaesthetic to obtain deep narcosis for all sizes of the fish. As shown in Fig 4, 200 mg L^-1^ metacaine give low induction time (Fig 2A) and recovery times (Fig 2B) and can therefore be used as anaesthetic for full anaesthesia to deep narcosis of lumpfish independent of fish size and temperature. The statistical analyses of effect of anaesthetic concentrations, temperature and fish size and the combined effects of those (Fig 4C), showed that each parameter separately and anaesthetic concentration in combination with temperature and fish size have a significant effect. However, there were no interactions between temperature and fish size, nor concentration, temperature and fish size (Fig 4C).

### Therapeutic window and conditions for euthanasia

**Fig 5:**
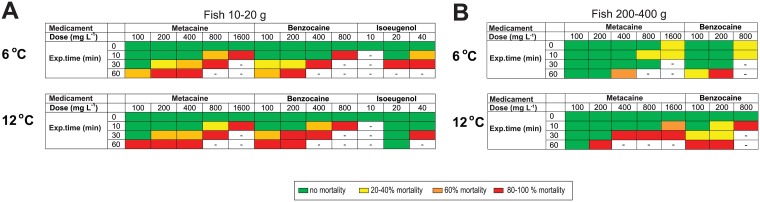
Therapeutic windows and conditions for lethal conditions. Therapeutic window for different anaesthetic concentrations and exposure times in different anaesthetic solutions (A) Effect of metacaine, benzocaine and isoeugenol on small fish (10–20 g) at 6°C (*upper panel*) and 12°C (*lower panel*). (B) Effect of metacaine and benzocaine on medium sized fish (200–400 g) at 6°C (*upper panel*) and 12°C (*lower panel*). Percentage mortality is indicated by colors: green = no mortality, yellow = 20–40% mortality, orange = 60% mortality, red = 80–100% mortality. N = 5.

Determination of therapeutic window was performed in order to ensure that the recommended anaesthetic concentrations had a good safety margin both with regard to concentration and exposure time. Based on pilot experiments, the exposure times were 10, 30 and 60 min (N = 5) for each dose and each drug at both 6°C and 12°C for all fish sizes (Fig 5). None of the lumpfish died after 0-exposure. For metacaine 200 mg L^-1^, small fish could be exposed to this dose for 10 minutes and medium sized fish could be exposed for 60 and 30 minutes at 6°C and 12°C, respectively. The largest fish were only exposed to the different concentrations for 10 minutes. Under these conditions, no mortality was observed. Based on our data, an overview of recommended concentrations for full anaesthesia for fish of different sizes and at different temperatures is given in Table 2.

**Table 2:** Overview of recommended concentrations for full anaesthesia of lumpfish.

Anaesthetic*	Fish size (g)	Temp (°C)	Concentration normal anaesthesia (mg L^-1^)	Max exposure time (min)	Concentration fast anaesthesia (mg L^-1^)	Max exposure time (min)
Metacaine	10–20	6	100	30	200	10
		12	100	30	200	10
	200–400	6	200	60	n.d.	n.d
		12	200	30	n.d.	n.d.
	600–1300	6	200	10^1^	n.d.	n.d
		12	200	10^1^	n.d.	n.d.
Benzocaine	10–20	6	100	10	200	10
		12	100	10	200	10
	200–400	6	Not recommended			
		12	100	10	200	< 10

*Isoeugenol is not recommended for full anaesthesia of lumpfish due to long recovery phase.

^1^ = the large fish (600-1300g) was not incubated for more than 10 minutes.

n.d. = good conditions for fast anaesthesia with regards to both acceptable induction and recovery times was not found.

## Discussion

### Criteria for monitoring depth of anaesthesia and response to external stimuli

Prior to this study there were no established protocols for full anaesthesia or euthanasia of lumpfish although it has been intensively farmed the last few years [2]. As lumpfish prefers to adhere to the substrate rather than to swim actively under normal conditions, and thus exhibit different behavioral elements and postures compares to other species like salmon and cod, an important first step in this study was to determine useful criteria to assess anaesthesia for lumpfish. An overview of behavior during different stages of anaesthesia in fish has been given by Zahl and co-workers [32], but this was not tailored for lumpfish and we had to modify it. For lumpfish, the excitatory behavior, defined by irregular or increased respiration and increased activity, was observed before the fish was disoriented and had reduced activity as described for other species [32]. This behavior was typically observed at high doses (> 400 mg L^-1^ metacaine). Following excitatory stage, lumpfish was sedated and had reduced activity, disorientation and loss of equilibrium. In stage III, there were no swimming activity, cessation of fin movement and the operculum movements were weak. When the operculum movements were nearly absent, the lumpfish were transferred to the recovery tank. This was defined as the 0-exposure. No fish died at this stage in the current study. However, it should be noticed that this is the end of the third plane of stage III [33], which is deep narcosis, and lighter anaesthesia will likely be sufficient for most task. Loss of equilibrium combined with end of swimming activity (Stage III, plane 1) can then be used as criteria, and can be obtained by for example 100 mg L^-1^ metacaine. Time measurements for this stage for metacaine, benzocaine and isoeugenol are also included in S1–S3 Tables respectively.

To evaluate the depth of anaesthesia, the lumpfish’s reaction to external stimuli was monitored. Pinch in the tail is commonly used to provoke a reaction in other fish species like zebrafish and Atlantic salmon [32]. This was however not a useable criterion for lumpfish as many of the fish showed no reaction to the pinch. The same lack of reaction was seen when pinpricking in the skin. This might be because lumpfish lack Mauthner neurons which initiate escape responses [34]. The best way to provoke a reaction in lumpfish was to pinch in the lower lip as this caused a bite reflex and this technique was used in this study to evaluate if the fish were anaesthetized or not. Lumpfish did not react to pinch in the lower lip at stage III, plane 1–3.

### Anaesthetic concentrations and behavior upon anaesthesia

For the current study, we got approval from the Norwegian Food authorities to develop protocols for full anaesthesia for lumpfish by use of metacaine, benzocaine and isoeugenol. Local federal or national legislation in other countries to use these anaesthetics on lumpfish will be needed to consider before treatments. Initial pilot experiments showed that the anaesthetic doses recommended for Atlantic salmon and rainbow trout, 50–60 mg L^-1^ for metacaine, 30–40 mg L^-1^ for benzocaine and 10–14 mg L^-1^ isoeugenol (www.felleskatalogen.no/medisin-vet), gave unsatisfactory long induction times for lumpfish and could therefore not be used. A new set of concentrations were therefore used in this study (100–1600 mg L^-1^ of metacaine, 100–800 mg L^-1^ of benzocaine and 10–40 mg L^-1^ of isoeugenol).

The initial reaction upon high concentration of metacaine was increased respiration rate and the fish were swimming toward the surface. Upon exposure to isoeugenol, faeces was often observed in the water and for many of the fish, the operculum movement did not stop, even after 20 min exposure. The recovery time after exposure to isoeugenol was in general very long and we found that isoeugenol could be used for full anaesthesia only for the smallest fish size, and even for these small fish, the recovery could be longer than one 1 hour at 6°C. The recovery time at 12°C was shorter and the individual variation within the fish group was less than at 6°C. Typical behavior when exposed to benzocaine was disoriented swimming pattern for several minutes before being anaesthetized. Benzocaine is suitable as anaesthetic for small and medium sized fish, although the recovery times for medium sized fish at 6°C was very long, even for the lowest doses.

### Effects of anaesthetic concentrations

This study also shows a clear relationship between dose and induction and recovery times where an increase in dose shortens the induction time and prolongs the recovery time. Critical drug concentrations are therefore reached more rapidly with higher anaesthetic exposure levels, thus supporting the hypothesis that simple diffusion and osmotic pressure are principally responsible for the uptake of anaesthetics over the gills.

The dosages of the agents used in the current study are higher that the doses recommended for Atlantic salmon but within the range of those reported as optimal for various fish species (see [12,16] for details). A light sedation with reduced response to external stimuli might be sufficient during practices such as transport, sorting and netting whereas surgery require anaesthetized fish with no responses or reflex reactions to the procedure. It has been reported that isoeugenol can be used for sedation of lumpfish [35]. Iversen and his coworkers also showed that the plasma cortisol level was lower in lumpfish compared to other fish species. Since lumpfish has a very different behavior compared with other fish species, and currently there is not defined good parameters to evaluate if/ at which level lumpfish is sedated, it could be considered if it is necessary to sedate lumpfish during transport and easy/quick handling procedures like sorting, netting and vaccination. This consideration is based on the long recovery times found in this study after exposure to even low concentrations of isoeugenol.

### Effect of sea water temperature

Our study showed that a rise in temperature from 6 to 12°C shortened both induction and recovery times for all the drugs, doses and weight classes tested. Shortened induction and recovery times with increasing temperature have also been reported for several other teleost species [30,36–39] and is most probably related to an increased basal metabolic rate which requires higher demand for oxygen leading to increased respiration and circulation [40–42].

### Effect of body size

Since metacaine is the only anaesthetic agent that was studied for all three weight groups, the importance of fish size on the anaesthetic effect is limited to that drug. In previous studies a number of anaesthetic agents have been examined regarding the importance of body weight and diverging results have been reported for various fish species [38, 43–47]. Some studies demonstrate no relationship between body size and induction and recovery time, whereas others suggest that such a relationship does exist. In the present study we found increased induction and recovery times with increasing weight of the fish at both 6°C and 12°C and at all doses. Atlantic cod and Atlantic halibut are examples of other species where increasing induction and recovery time with increasing weight are reported [30, 33, 38]. This suggests that the rate of absorption of the anaesthetic in relation to weight is slower in larger fish and may be a reflection of the smaller gill surface area in relation to body mass as a smaller area is available for drug diffusion relative to size.

### Pharmacokinetic

The effect of anaesthetic agents on lumpfish is in this study visualized by induction and recovery times and where the recovery is divided in three phases, namely initial respiration, normal respiration and swimming activity. Induction is directly depending on pharmacokinetic properties of the compounds and as no studies are available that measures the absorption kinetics of anaesthetics in fish in terms of plasma concentrations, the induction time is used to describe this process and where a short induction time indicates a fast absorption rate. The absorption rates of these compounds are slower in lumpfish than in Atlantic salmon as the initial concentration tested were too low to induce anaesthetic effect in lumpfish within an acceptable time period. One factor that can explain this is if the gill surface area in relation to body weight is smaller in lumpfish compared to more active species like Atlantic salmon and Atlantic cod (*Gadus morhua* L.). Furthermore, lower activity is followed by lesser requirement for oxygen, giving slower gill movements, another aspect that may influence the absorption rate. Induction times were found to be longer for benzocaine and metacaine in lumpfish compared with Atlantic salmon, Atlantic cod and Atlantic halibut (*Hippoglossus hippoglossus* L.) even though the doses used for these species were lower [30, 33, 48]. An exception was a dose of 40 mg L^-1^ of isoeugenol applied for anaesthetising Atlantic halibut of 33 g at a temperature of 8°C, that gave a longer induction time (2.3 min) [33] compared to lumpfish (10 g) at 6°C with an induction time of 1.5 min.

There are only a few studies presenting data on the elimination kinetics of anaesthetics in fish. This pharmacokinetic process is therefore often described for anaesthetics in fish by the recovery period and where a long recovery period indicates a slow clearance process. In Atlantic salmon the plasma clearance rates of isoeugenol, benzocaine and metacaine were found to be 0.059, 0.35 and 3.10 l kg−1 h−1 respectively [48]. These clearance rates show that Atlantic salmon need longer time to recover from benzocaine and especially isoeugenol anaesthesia than from metacaine anaesthesia. These observations are similar to what we found in the present study. The longest recovery times where observed after exposure to isoeugenol, especially at the low temperature, followed by benzocaine. The recovery times upon exposure to metacaine were much shorter, although at high concentrations (800–1600 mg L^-1^) and low temperature, the recovery time could be long even for this drug. Due to lower activity in general of lumpfish it was likely to believe that the elimination of anaesthetics would be slower compared with more active species like Atlantic cod. This study showed however that the recovery times of metacaine and benzocaine with 2.7 and 3.5 min respectively in lumpfish (10 g, 6°C) were similar to the values found for Atlantic cod (10 g, 8°C) with 3.78 and 3.56 min respectively and much shorter than in Atlantic halibut (33 g, 8°C) with 12.4 and 19.3 min respectively [30, 33]. In comparison, isoeugenol the most lipophilic of the tested compounds, was very slowly eliminated from lumpfish with a recovery time of 56.3 min (10 g, 6°C) which was twice the recovery time found in Atlantic halibut with 26.2 min (33 g, 8°C) [33].

### Euthanasia

An overdose of anaesthetic drugs is commonly used to kill fish, but care should be taken to maintain fish welfare [22]. In order for an overdose of anaesthetic to be a reliable and humane killing method for fish, more knowledge is needed before recommending the minimum dosage and exposure times for specific life stages, body sizes and water temperatures. Such information would help to ensure a minimum time to loss of consciousness and minimum induction of stress (EFSA, 2009a). If a fish has to be killed, then death must occur with the least possible anxiety, pain and distress. Metacaine offers an alternative to other means of chemical or physical fish euthanasia, but is not permitted for use prior to slaughter for any purpose on any fish that might enter the food chain (EFSA, 2004). A concentration of 250 mg L^-1^is the minimal concentration suggested by the AVMA for euthanasia of amphibians and fish (AVMA, 2007). A lethal concentration of 400–500 mg L^-1^ is generally used for euthanasia of salmonids [10]. For lumpfish metacaine and benzocaine could be used for euthanasia for small fish (at 6°C and 12°C) and medium sized fish (12°C) whereas the concentration must be increased to 1600 mg L^-1^ and 800 mg L^-1^ for metacaine and benzocaine, respectively, in order to obtain safe euthanasia as shown in Fig 5. For medium sized fish, at 6°C, none of the tested conditions were suitable for euthanasia.

## Conclusions

The results in our study suggest that metacaine is the most optimal anaesthetic for lumpfish. A concentration of 200 mg L^-1^ of metacaine is efficient to obtain deep narcosis independent of fish size (10–1300 g) and sea water temperatures in the range 6–12°C, and that the safety margins regarding dose and exposure time appear sound. Isoeugenol is not recommended for full anaesthesia due to prolong induction and recovery times. Benzocaine can be used for small fish and medium sized fish at 12°C, but not at 6°C. An overview of recommended doses for full anaesthesia of lumpfish is given in Table 2. Conditions for euthanasia are summarized in Fig 5. In the current study we have determined conditions for deep narcosis, but lighter anaesthesia may be sufficient for most tasks. This can be obtained with 100 mg L^-1^ metacaine. At that stage, the lumpfish has stopped swimming, but do not react to externa stimuli. Further pharmacokinetic and physiological studies should however be performed to determine if anaesthesia of lumpfish has any adverse long-term effect. Before such information is available, it is debatable whether full anaesthesia is necessary for quick handling procedures such as vaccination and visible implant elastomer tagging. Guidelines for efficient anaesthetic concentrations for lumpfish of different sizes at different sea water temperatures are important for good animal well-fare and safe handling of the fish.

## Supporting information

S1 Table Induction and recovery times for all fish anaesthetized with metacaine. (DOCX)

S2 Table Induction and recovery times for all fish anaesthetized with benzocaine. (DOCX)

S3 Table Induction and recovery times for all fish anaesthetized with isoeugenol. (DOCX)

S4 Table Statistical analyses to Figs 1–3 summarizing the effect of concentrations on induction time (deep narcosis) and effect of temperature. (DOCX)

S5 Table Statistical analyses to Figs 1–3 summarizing the effect of concentrations on initial respiration time and effect of temperature. (DOCX)

S6 Table Statistical analyses to Figs 1–3 summarizing the effect of concentrations on normal respiration time and effect of temperature. (DOCX)

S7 Table Statistical analyses to Figs 1–3 summarizing the effect of concentrations on swimming time and effect of temperature. (DOCX)

## References

[1] Imsland AK, Reynolds P, Eliassen G, Hangstad TA, Foss A, Vikingstad E, et al The use of lumpfish (*Cyclopterus lumpus* L) to control sea lice (*Lepeophtheirus salmonis* Krøyer) infestations in intensively farmed Atlantic salmon (*Salmo salar* L). Aquaculture. 2014;424:18–23. doi: 10.1016/j.aquaculture.2013.12.033

[2] Powell A, Treasurer JW, Pooley CL, Keay AJ, Llioyd R, Imsland AK, et al Use of lumpfish for sea-lice control in salmon farming: challenges and opportunities. Reviews in Aquaculture. 2017;1–20. doi: 10.1111/raq.12194

[3] Barton BA. Stress in fishes: a diversity of responses with particular reference to changes in circulating corticosteroids. Integr Comp Biol. 2002;42:517–525. doi: 10.1093/icb/42.3.517 . 21708747 10.1093/icb/42.3.517

[4] Bonga SEW. The stress response in fish. Physiol Rev. 1997;77(3):591–625. 9234959 10.1152/physrev.1997.77.3.591

[5] Braithwaite VA, Huntingford FA. Fish and welfare: do fish have the capacity for pain perception and suffering? Anim Welfare. 2004;13:S87–S92.

[6] Rose JD. The Neurobehavioral nature of fishes and the question of awareness and pain. Rev Fish Sci. 2002;10:1–38. doi: 10.1080/20026491051668

[7] Sneddon LU. Anatomical and electrophysiological analysis of the trigeminal nerve in a teleost fish, *Oncorhynchus mykiss*. Neurosci Lett. 2002;319:167–171. 11834319 10.1016/s0304-3940(01)02584-8

[8] Sneddon LU. The evidence for pain in fish: the use of morphine as an analgesic. Appl Anim Behav Sci. 2003;83:153–162.

[9] Sneddon LU. Trigeminal somatosensory innervation of the head of a teleost fish with particular reference to nociception. Brain Res. 2003;972:44–52. 12711077 10.1016/s0006-8993(03)02483-1

[10] Dunlop R, Laming P. Mechanoreceptive and nociceptive responses in the central nervous system of goldfish (*Carassius auratus*) and trout (*Oncorhynchus mykiss*). J Pain. 2005;6:561–568. doi: 10.1016/j.jpain.2005.02.010 16139775 10.1016/j.jpain.2005.02.010

[11] Chandroo KP, Duncan IJH, Moccia RD. Can fish suffer?: perspectives on sentience, pain, fear and stress. Appl Anim Behav Sci. 2004;86:225–250.

[12] Ackerman PA, Morgan JD, Iwama GK. Anesthetics. CCAC guidelines on: the care and use of fish in research, teaching and testing. 2005. http://www.ccac.ca/en/CCAC_Programs/Guidelines_Policies/GDLINES/Fish/Fish.

[13] Javahery S, Nekoubin H, Moradlu AH. Effect of anaesthesia with clove oil in fish (review). Fish Physiol Biochem. 2012;38:1545–1552. doi: 10.1007/s10695-012-9682-5 22752268 10.1007/s10695-012-9682-5

[14] Neiffer DL, Stamper MA. Fish Sedation, Anesthesia, Analgesia, and Euthanasia: Considerations, Methods, and Types of Drugs. Ilar J. 2009;50:343–360. 19949251 10.1093/ilar.50.4.343

[15] Popovic NT, Strunjak-Perovic I, Coz-Rakovac R, Barisic J, Jadan M, Berakovic AP, et al Tricaine methane-sulfonate (MS-222) application in fish anaesthesia. J Appl Ichthyol. 2012;28:553–64.

[16] Ross LG, Ross B. Anaesthetic and sedative techniques for aquatic animals. Ross LG, Ross B, editors. Oxford, UK: Blackwell; 2008.

[17] Frazier DT, Narahashi T. Tricaine (Ms-222)—Effects on Ionic Conductances of Squid Axon-Membranes. Eur J Pharmacol. 1975;33:313–317. 171163 10.1016/0014-2999(75)90175-2

[18] Neumcke B, Schwarz W, Stampfli R. Block of Na Channels in the Membrane of Myelinated Nerve by Benzocaine. Pflug Arch Eur J Phy. 1981;390:230–6. 10.1007/BF00658267 6265861

[19] Hara K, Sata T. The effects of the local Anesthetics lidocaine and procaine on glycine and gamma-aminobutyric acid receptors expressed in xenopus oocytes. Anesth Analg. 2007;104:1434–9. doi: 10.1213/01.ane.0000261509.72234.a6 17513637 10.1213/01.ane.0000261509.72234.a6

[20] Hedrick MS, Winmill RE. Excitatory and inhibitory effects of tricaine (MS-222) on fictive breathing in isolated bullfrog brain stem. Am J Physiol-Reg I. 2003;284:R405–R412. 10.1152/ajpregu.00418.2002 12414435

[21] Ueta K, Suzuki T, Sugimoto M, Uchida I, Mashimo T. Local Anesthetics have different mechanisms and sites of action at recombinant 5-HT3 receptors. Region Anesth Pain M. 2007;32:462–470. 10.1016/j.rapm.2007.06.391 18035290

[22] Close B, Banister K, Baumans V, Bernoth EM, Bromage N, Bunyan J, et al Recommendations for euthanasia of experimental animals. Part 2. Lab Anim. 1997;31:1–32. doi: 10.1258/002367797780600297 9121105 10.1258/002367797780600297

[23] Aoshima H, Hamamoto K. Potentiation of GABAA receptors expressed in Xenopus oocytes by perfume and phytoncid. Biosci Biotechnol Biochem. 1999;63:743–748. . 10361687 10.1271/bbb.63.743

[24] Lee MH, Yeon KY, Park CK, Li HY, Fang Z, Kim MS, et al Eugenol inhibits calcium currents in dental afferent neurons. J Dent Res. 2005;84:848–851. doi: 10.1177/154405910508400913 16109996 10.1177/154405910508400913

[25] Park CK, Li HY, Yeon KY, Jung SJ, Choi SY, Lee SJ, et al Eugenol inhibits sodium currents in dental afferent neurons. J Dent Res. 2006;85:900–904. doi: 10.1177/154405910608501005 16998128 10.1177/154405910608501005

[26] Wie MB, Won MH, Lee KH, Shin JH, Lee JC, Suh HW, et al Eugenol protects neuronal cells from excitotoxic and oxidative injury in primary cortical cultures. Neurosci Lett. 1997;225:93–96. 9147382 10.1016/s0304-3940(97)00195-x

[27] Bell G. An outline of anesthetics and anesthesia for salmonids: a guide for fish culturists in British Columbia. Canadian technical report of fisheries aquatic sciences no. 15341987. p. 16.

[28] Marking LL, Meyer FP. Are Better Anesthetics Needed in Fisheries. Fisheries. 1985;10:2–5.

[29] Kristan J, Stara A, Polgesek M, Drasovean A, Kolarova J, Priborsky J, et al Efficacy of different anaesthetics for pikeperch (*Sander lucioperca* L.) in relation to water temperature. Neuroendocrinol Lett. 2014;35:81–85. 25638370

[30] Zahl IH, Kiessling A, Samuelsen OB, Hansen MK. Anaesthesia of Atlantic cod (*Gadus morhua*)—Effect of pre-anaesthetic sedation, and importance of body weight, temperature and stress. Aquaculture. 2009;295:52–59.

[31] Zahl IH, Kiessling A, Samuelsen OB, Olsen RE. Anesthesia induces stress in Atlantic salmon (*Salmo salar*), Atlantic cod (*Gadus morhua*) and Atlantic halibut (*Hippoglossus hippoglossus*). Fish Physiol Biochem. 2010;36:719–730. doi: 10.1007/s10695-009-9346-2 19680764 10.1007/s10695-009-9346-2

[32] Zahl IH, Samuelsen O, Kiessling A. Anaesthesia of farmed fish: implications for welfare. Fish Physiol Biochem. 2012;38:201–218. doi: 10.1007/s10695-011-9565-1 22160749 10.1007/s10695-011-9565-1

[33] Zahl IH, Kiessling A, Samuelsen OB, Hansen MK. Anaesthesia of Atlantic halibut (*Hippoglossus hippoglossus*)—Effect of pre-anaesthetic sedation, and importance of body weight and water temperature. Aquac Res. 2011;42:1235–45.

[34] Hale ME. Fast start behaviors of fish lacking Mauthner neurons. Am Zool. 2000;40:1040–1041.

[35] Iversen MH, Jakobsen R, Eliassen RA, Ottesen O. Sedasjon av berggylte og rognkjeks for å redusere stress og dødelighet. nfexpert BIOLOGI-SVINN. 2014:42–46.

[36] Bowser PR. Anesthetic options for fish In: Gleed RD, Ludders J.W., editors. Recent advances in veterinary anesthesia and analgesia: companion animals. Ithaca, NY, USA: International Veterinary Information Service 2001.

[37] Hikasa Y, Takase K, Ogasawara T, Ogasawara S. Anesthesia and Recovery with Tricaine Methanesulfonate, Eugenol and Thiopental Sodium in the Carp, *Cyprinus-Carpio*. Jpn J Vet Sci. 1986;48:341–351. 10.1292/jvms1939.48.341 3712895

[38] Houston AH, Woods RJ. Influence of Temperature Upon Tricaine Methane Sulfonate Uptake and Induction of Anesthesia in Rainbow-Trout (*Salmo-Gairdneri*). Comp Biochem Phys C. 1976;54:1–6. 10.1016/0306-4492(76)90016-2 6200

[39] Sylvester JR, Holland LE. Influence of Temperature, Water Hardness, and Stocking Density on Ms-222 Response in 3 Species of Fish. Prog Fish Cult. 1982;44:138–141.

[40] Clarke A, Johnston NM. Scaling of metabolic rate with body mass and temperature in teleost fish. Journal of Americal Ecoloy. 1999;68:893–905.

[41] Saunders RL. Respiration of the Atlantic Cod. J Fish Res Board Can. 1963;20:373–386.

[42] Schurmann H, Steffensen JF. Effects of temperature, hypoxia and activity on the metabolism of juvenile Atlantic cod. J Fish Biol. 1997;50:1166–80.

[43] Gilderhus PA, Marking LL. Comparative efficacy of 16 anesthetic chemicals on rainbow trout. North Am J Fish Mana. 1987;7:288–292.

[44] Hoskonen P, Pirhonen J. Temperature effects on anaesthesia with clove oil in six temperate-zone fishes. J Fish Biol. 2004;64:1136–42.

[45] Olsen YA, Einarsdottir IE, Nilssen KJ. Metomidate Anesthesia in Atlantic Salmon, Salmo-Salar, Prevents Plasma-Cortisol Increase during Stress. Aquaculture. 1995;134:155–68.

[46] Stehly GR, Gingerich WH. Evaluation of AQUI-S (TM) (efficacy and minimum toxic concentration) as a fish anaesthetic sedative for public aquaculture in the United States. Aquac Res. 1999;30(5):365–372.

[47] Tsantilas H, Galatos AD, Athanassopoulou F, Prassinos NN, Kousoulaki K. Efficacy of 2-phenoxyethanol as an anaesthetic for two size classes of white sea bream, *Diplodus sargus* L., and sharp snout sea bream, *Diplodus puntazzo* C. Aquaculture. 2006;253:64–70.

[48] Kiessling A, Johansson D, Zahl IH, Samuelsen OB. Pharmacokinetics, plasma cortisol and effectiveness of benzocaine, MS-222 and isoeugenol measured in individual dorsal aorta-cannulated Atlantic salmon (*Salmo salar*) following bath administration. Aquaculture. 2009;286:301–8.

