# Supplementary material for: Development of anaesthetic protocols for lumpfish (Cyclopterus lumpus L.): Effect of anaesthetic concentrations, sea water temperature and body weight
Source: PLoS One. 2017 Jul 5;12(7):e0179344. doi: 10.1371/journal.pone.0179344 (PMC5497946; doi:10.1371/journal.pone.0179344)
Supplement: S1 Table — (DOCX) [file pone.0179344.s001.docx]

**S1 Table. Induction and recovery times for all fish anaesthetized with metacaine**

|  |  |  |  | **Metacaine (mg L^-1^)** (average time (minutes) ± S.D. N = 10 for small and medium sized fish N = 5 for large fish) | | | | |
| --- | --- | --- | --- | --- | --- | --- | --- | --- |
| **Phase** | **Activity** | **Fish size (g)** | **Temp (**°C) | **100** | **200** | **400** | **800** | **1600** |
| INDUCTION | No swimming | 10-20 | 6 | 2.1 ± 0.5 | 0.9 ± 0.1 | 0.6 ± 0.1 | 0.5 ± 0.1 | 0.3 ± 0.1 |
|  |  |  | 12 | 1.2 ± 0.3 | 0.5 ± 0.1 | 0.3 ± 0.1 | 0.2 ± 0.1 | 0.2 ± 0.1 |
|  |  | 200-400 | 6 | 4.1 ± 1.6 | 1.7 ± 0.6 | 1.1 ± 0.2 | 1.3 ± 0.4 | 1.0 ± 0.3 |
|  |  |  | 12 | 3.0 ± 0.9 | 1.2 ± 0.6 | 0.5 ± 0.2 | 0.4 ± 0.1 | 0.3 ± 0.1 |
|  |  | 600-1300 | 6 | 10.5 ± 1.3 | 4.0 ± 0.7 | 1.8 ± 0.3 | n.d. | n.d. |
|  |  |  | 12 | 4.5 ± 0.8 | 1.8 ± 0.4 | 1.4 ± 0.3 | n.d. | n.d. |
|  | No respiration | 10-20 | 6 | 5.0 ± 0.9 | 1.9 ± 0.5 | 0.8 ± 0.2 | 0.6 ± 0.1 | 0.4 ± 0.1 |
|  |  |  | 12 | 3.6 ± 0.9 | 1.2 ± 0.3 | 0.4 ± 0.1 | 0.3 ± 0.1 | 0.3 ± 0.0 |
|  |  | 200-400 | 6 | 8.4 ± 6.4 | 3.9 ± 0.7 | 2.5 ± 1.1 | 2.3 ± 0.7 | 1.5 ± 0.3 |
|  |  |  | 12 | 6.7 ± 1.0 | 3.5 ± 1.0 | 1.8 ± 0.5 | 0.6 ± 0.2 | 0.5 ± 0.1 |
|  |  | 600-1300 | 6 | 18.5 ± 1.5 | 6.7 ± 1.4 | 5.1 ± 1.2 | n.d. | n.d. |
|  |  |  | 12 | 6.3 ± 0.4 | 4.5 ± 1.1 | 2.0 ± 0.3 | n.d. | n.d. |
| RECOVERY | Initial respiration | 10-20 | 6 | 0.1 ± 0.1 | 0.5 ± 0.4 | 2.0 ± 2.0 | 5.2 ± 2.1 | 9.3 ± 1.6 |
|  |  |  | 12 | 0.2 ± 0.2 | 0.2 ± 0.1 | 0.8 ± 0.4 | 4.0 ± 1.4 | 5.4 ± 0.5 |
|  |  | 200-400 | 6 | 1.3 ± 1.7 | 2.3 ± 1.5 | 6.4 ±3.5 | 16.6 ± 4.2 | 17.8 ± 12.3 |
|  |  |  | 12 | 0.1 ± 0.1 | 0.9 ± 1.0 | 1.8 ± 0.9 | 6.5 ±2.5 | 7.7 ± 2.3 |
|  |  | 600-1300 | 6 | 0.1 ± 0.1 | 1.4 ± 1.6 | 4.4 ± 2.9 | n.d. | n.d. |
|  |  |  | 12 | 0.8 ± 0.7 | 1.5 ± 0.6 | 7.0 ± 6.5 | n.d. | n.d. |
|  | Normal respiration | 10-20 | 6 | 0.6 ± 0.2 | 1.6 ± 1.5 | 4.1 ± 3.2 | 9.5 ± 3.4 | 14.3 ± 2.1 |
|  |  |  | 12 | 0.4 ± 0.3 | 0.4 ± 0.1 | 1.3 ± 0.8 | 5.4 ± 1.8 | 10.9 ± 1.8 |
|  |  | 200-400 | 6 | 3.4 ± 3.3 | 5.7 ± 2.1 | 11.3 ± 4.7 | 27.8 ± 7.6 | 26.1 ± 12.5 |
|  |  |  | 12 | 1.0 ± 1.1 | 4.2 ± 3.4 | 4.4 ± 2.0 | 9.1 ± 4.2 | 14.5 ± 4.2 |
|  |  | 600-1300 | 6 | 3.4 ± 2.3 | 6.9 ± 3.1 | 23.8 ± 6.2 | n.d. | n.d. |
|  |  |  | 12 | 3.8 ± 3.9 | 8.0 ± 3.6 | 17.6 ± 6.7 | n.d. | n.d. |
|  | Swimming | 10-20 | 6 | 1.5 ± 0.4 | 2.7 ± 1.7 | 5.3 ± 3.0 | 11.2 ± 3.3 | 15.9 ± 2.6 |
|  |  |  | 12 | 1.0 ± 0.7 | 1.1 ± 0.4 | 2.2 ±0.9 | 6.8 ± 2.1 | 12.6 ± 2.4 |
|  |  | 200-400 | 6 | 4.8 ± 3.6 | 7.2 ± 2.4 | 13.4 ± 5.1 | 32.3 ± 8.5 | 30.0 ± 15.2 |
|  |  |  | 12 | 2.0 ± 1.3 | 5.9 ± 4.0 | 5.4 ± 2.0 | 10.5 ± 4.8 | 16.3 ± 4.6 |
|  |  | 600-1300 | 6 | 5.6 ± 2.9 | 9.6 ± 3.8 | 29.0 ± 7.2 | n.d. | n.d. |
|  |  |  | 12 | 5.5 ± 4.1 | 9.8 ± 4.2 | 21.0 ± 7.7 | n.d. | n.d. |

n.d. = not determined
