# Supplementary material for: Development of anaesthetic protocols for lumpfish (Cyclopterus lumpus L.): Effect of anaesthetic concentrations, sea water temperature and body weight
Source: PLoS One. 2017 Jul 5;12(7):e0179344. doi: 10.1371/journal.pone.0179344 (PMC5497946; doi:10.1371/journal.pone.0179344)
Supplement: S2 Table — (DOCX) [file pone.0179344.s002.docx]

**S2 Table. Induction and recovery times for all fish anaesthetized with benzocaine**

|  |  |  |  | **Benzocaine (mg L^-1^)** (average time (minutes) ± S.D.,  N = 10 for small and medium sized fish N = 5 for large fish) | | | |
| --- | --- | --- | --- | --- | --- | --- | --- |
| **Phase** | **Activity** | **Fish size (g)** | **Temp (**°C) | **100** | **200** | **400** | **800** |
| INDUCTION | No swimming | 10-20 | 6 | 2.9 ± 1.3 | 1.4 ± 0.3 | 0.9 ± 0.2 | 0.4 ± 0.1 |
|  |  |  | 12 | 1.3 ± 0.4 | 0.6 ± 0.1 | 0.3 ± 0.1 | 0.2 ± 0.0 |
|  |  | 200-400 | 6 | 5.5 ± 1.8 | 2.2 ± 0.9 | n.d. | 1.3 ± 0.5 |
|  |  |  | 12 | 2.6 ± 0.8 | 0.9 ± 0.3 | n.d. | 0.5 ± 0.1 |
|  | No respiration | 10-20 | 6 | 4.5 ± 2.0 | 2.2 ± 0.6 | 1.2 ± 0.3 | 0.5 ± 0.1 |
|  |  |  | 12 | 2.4 ± 0.7 | 1.1 ± 0.3 | 0.4 ± 0.1 | 0.3 ± 0.0 |
|  |  | 200-400 | 6 | 6.5 ± 1.9 | 4.0 ± 0.5 | n.d. | 1.9 ± 0.7 |
|  |  |  | 12 | 5.7 ± 5.1 | 1.6 ± 0.4 | n.d. | 0.6 ± 0.1 |
| RECOVERY | Initial respiration | 10-20 | 6 | 0.4 ± 0.6 | 1.2 ± 1.8 | 2.3 ± 1.5 | 7.1 ± 2.8 |
|  |  |  | 12 | 0.15 ± 0.1 | 0.6 ± 0.4 | 2.0 ± 0.8 | 6.3 ± 2.3 |
|  |  | 200-400 | 6 | 3.8 ± 2.7 | 7.9 ± 4.1 | n.d. | 18.1 ± 6.7 |
|  |  |  | 12 | 1.0 ± 1.4 | 1.8 ± 1.2 | n.d. | 9.2 ± 2.5 |
|  | Normal respiration | 10-20 | 6 | 1.1 ± 1.3 | 1.9 ± 2.2 | 5.2 ± 2.1 | 11.9 ± 3.8 |
|  |  |  | 12 | 1.7 ± 1.6 | 2.5 ± 1.3 | 4.8 ± 1.0 | 11.7 ± 4.6 |
|  |  | 200-400 | 6 | 14.1 ± 6.1 | 20.8 ± 7.2 | n.d. | 26.3 ± 8.1 |
|  |  |  | 12 | 6.8 ± 3.4 | 4.6 ± 1.0 | n.d. | 14.8 ± 4.3 |
|  | Swimming | 10-20 | 6 | 2.3 ± 1.5 | 4.1 ± 3.8 | 6.6 ± 2.1 | 13.2 ± 4.1 |
|  |  |  | 12 | 2.4 ± 2.0 | 3.4 ± 1.4 | 5.9 ± 1.0 | 14.0 ± 6.0 |
|  |  | 200-400 | 6 | 16.5 ± 6.5 | 24.1 ± 8.0 | n.d. | 33.7 ± 12.1 |
|  |  |  | 12 | 9.5 ± 3.6 | 5.5 ± 1.0 | n.d. | 17.1 ± 5.7 |

n.d. = not determined
