# Supplementary material for: Development of anaesthetic protocols for lumpfish (Cyclopterus lumpus L.): Effect of anaesthetic concentrations, sea water temperature and body weight
Source: PLoS One. 2017 Jul 5;12(7):e0179344. doi: 10.1371/journal.pone.0179344 (PMC5497946; doi:10.1371/journal.pone.0179344)
Supplement: S3 Table — (DOCX) [file pone.0179344.s003.docx]

**S3 Table. Induction and recovery times for all fish anaesthetized with isoeugenol**

|  |  |  |  | **Isoeugenol (mg L^-1^)** (average time (minutes) ± S.D. N = 10) | | |
| --- | --- | --- | --- | --- | --- | --- |
| **Phase** | **Activity** | **Fish size (g)** | **Temp (**°C) | **10** | **20** | **40** |
| INDUCTION | No swimming | 10-20 | 6 | 1.8 ± 0.3 | 1.3 ± 0.2 | 0.9 ±0.1 |
|  |  |  | 12 | 1.2 ± 0.3 | 0.8 ± 0.3 | 0.5 ± 0.1 |
|  | No respiration |  | 6 | 14.1 ± 2.2 | 7.1 ± 3.1 | 1.5 ± 0.5 |
|  |  |  | 12 | 6.5 ± 2.0 | 4.5 ± 1.3 | 1.0 ± 0.1 |
| RECOVERY | Initial respiration |  | 6 | 0.5 ± 0.7 | 0.6 ± 0.4 | 0.1 ± 0.4 |
|  |  |  | 12 | 1.0 ± 1.1 | 0.3 ± 0.2 | 1.6 ± 1.5 |
|  | Normal respiration |  | 6 | 0.7 ± 4.5 | 5.4 ± 4.3 | 20.7 ± 8.9 |
|  |  |  | 12 | 7.2 ± 5.5 | 7.5 ± 9.8 | 12.0 ± 6.9 |
|  | Swimming |  | 6 | 25.0 ± 8.8 | 24.1 ± 10.1 | 55.6 ± 22.2 |
|  |  |  | 12 | 17.4 ± 6.4 | 18.7 ± 15.1 | 24.5 ± 8.6 |
