# Supplementary material for: Development of anaesthetic protocols for lumpfish (Cyclopterus lumpus L.): Effect of anaesthetic concentrations, sea water temperature and body weight
Source: PLoS One. 2017 Jul 5;12(7):e0179344. doi: 10.1371/journal.pone.0179344 (PMC5497946; doi:10.1371/journal.pone.0179344)
Supplement: S4 Table — (DOCX) [file pone.0179344.s004.docx]

**S4 Table.** **Statistical analyses to Figure 1-3 summarizing the effect of concentrations on induction time (deep narcosis) and effect of temperature.**

| **Corresponding Figure** | **Stat test^1^** | **Fish Size (g)** | **Anaesthetic** | **Temp (°C)** | **Induction time** | | | | | **Significance** |
| --- | --- | --- | --- | --- | --- | --- | --- | --- | --- | --- |
|  |  |  |  |  | **Anaesthetic concentration (mg L^-1^)** | | | | |  |
|  |  |  |  |  | **10** | **20** | **40** |  |  |  |
| 1C | KW (D) | 10-20 | Isoeugenol | 6 | a | ab | c |  |  | H_2_ = 18.732 (P < 0.001) |
| 1F | KW (T) | 10-20 | Isoeugenol | 12 | a | ab | b |  |  | H_2_ = 15.616 (P < 0.001) |
| 1C vs 1F | MWRST | 10-20 | Isoeugenol | 6 vs 12 | T = 61 P = 0.012 | n.s. | T = 138 P = 0.014 |  |  |  |
|  |  |  |  |  | **100** | **200** | **400** | **800** | **1600** |  |
| 1A | 1wA (HS) | 10-20 | Metacaine | 6 | a | b | c | c | c | F_4,49_ = 157.734 (P < 0.001) |
| 1D | 1wA (HS) | 10-20 | Metacaine | 12 | a | b | c | c | c | F_4,49_ = 110.015 (P < 0.001) |
| 1A vs 1D | MWRST | 10-20 | Metacaine | 6 vs 12 | T = 142 P = 0.006 | T = 144 P = 0.004 | T= 147.5 P = 0.001 | T = 155 P < 0.001 | T = 146.5 P = 0.002 |  |
| 1B | KW (T) | 10-20 | Benzocaine | 6 | a | ab | bc | c | n.d. | H_3_ = 36.161 (P < 0.001) |
| 1E | KW (T) | 10-20 | Benzocaine | 12 | a | ab | bc | c | n.d. | H_3_ = 34.458 (P < 0.001) |
| 1B vs 1E | MWRST | 10-20 | Benzocaine | 6 vs 12 | T = 151.5 P < 0.001 | T = 153.5 P < 0.001 | T = 155 P < 0.001 | T = 155 P < 0.001 | n.d. |  |
| 2A | KW (T) | 200-400 | Metacaine | 6 | a | ab | bc | c | c | H_4_ = 35.1 (P < 0.001) |
| 2C | KW (T) | 200-400 | Metacaine | 12 | a | ab | bc | cd | d | H_4_ = 45.587 (P < 0.001) |
| 2A vs 2C | MWRST | 200-400 | Metacaine | 6 vs 12 | n.s. | n.s. | n.s. | T = 155 P < 0.001 | T = 155 P < 0.001 |  |
| 2B | KW (T) | 200-400 | Benzocaine | 6 | a | b | n.d | c | n.d. | H_2_ = 24.050 (P < 0.001) |
| 2D | KW (T) | 200-400 | Benzocaine | 12 | a | b | n.d. | c | n.d. | H_2_ = 25.835 (P < 0.001) |
| 2B vs 2D | MWRST | 200-400 | Benzocaine | 6 vs 12 | T= 139 P = 0.011 | T= 155 P < 0.001 | n.d. | T = 155 P < 0.001 | n.d. |  |
| 3A | 1wA (HS) | 600-1300 | Metacaine | 6 | a | b | b | n.d. | n.d. | F_2,14_ = 138.061 (P < 0.001) |
| 3B | 1wA (HS) | 600-1300 | Metacaine | 12 | a | b | c | n.d. | n.d. | F_2,14_ = 50.742 (P < 0.001) |
| 3A vs 3B | MWRST | 600-1300 | Metacaine | 6 vs 12 | T = 40 P = 0.008 | T = 38 P = 0.032 | T = 40 P = 0.008 | n.d. | n.d. |  |

**^1^**The post hoc tests are given in the parenthesis, D = Dunn’s method , T= Tukey test, HS= Holm-Sidak method

* To evaluate differences between temperatures, Mann-Whitney Rank Sum Test (MWRST) was performed

n.s.= not significant

n.d. = not determined (not included in the study)
