# Supplementary material for: Development of anaesthetic protocols for lumpfish (Cyclopterus lumpus L.): Effect of anaesthetic concentrations, sea water temperature and body weight
Source: PLoS One. 2017 Jul 5;12(7):e0179344. doi: 10.1371/journal.pone.0179344 (PMC5497946; doi:10.1371/journal.pone.0179344)
Supplement: S5 Table — (DOCX) [file pone.0179344.s005.docx]

**S5 Table. Statistical analyses to Figure 1-3 summarizing the effect of anaesthetic concentrations for initial respiration time and effect of temperature.**

| **Corresponding Figure** | **Stat test^1*^** | **Fish size (g)** | **Anaesthetic** | **Temp (°C)** | **Induction time** | | | | | **Significance** |
| --- | --- | --- | --- | --- | --- | --- | --- | --- | --- | --- |
|  |  |  |  |  | **Doses (mg L^-1^)** | | | | |  |
|  |  |  |  |  | **10** | **20** | **40** |  |  |  |
| 1C | KW (D) | 10-20 | Isoeugenol | 6 | ab | b | a |  |  | H_2_ = 9.586 (P < 0.008) |
| 1F | KW (T) | 10-20 | Isoeugenol | 12 | a | b | a |  |  | H_2_ = 20.373 (P < 0.001) |
| 1C vs 1F | MWRST | 10-20 | Isoeugenol | 6 vs 12 | T = 22 P = 0.032 | T = 132 P = 0.045 | T=134 P = 0.031 |  |  |  |
|  |  |  |  |  |  |  |  |  |  |  |
|  |  |  |  |  | **100** | **200** | **400** | **800** | **1600** |  |
| 1A | KW (T) | 10-20 | Metacaine | 6 | d | cd | bc | ab | a | H_4_ = 43.109 (P < 00.1) |
| 1D | KW (T) | 10-20 | Metacaine | 12 | c | c | bc | ab | a | H_4_ = 42.234 (P < 00.1) |
| 1A vs 1D | MWRST | 10-20 | Metacaine | 6 vs 12 | n.s. | T = 144 P = 0.004 | n.s. | n.s. | T = 154 P < 0.001 |  |
| 1B | KW (T) | 10-20 | Benzocaine | 6 | b | b | ab | a | n.d. | H_3_=25.929 (P < 0.001) |
| 1E | KW (T) | 10-20 | Benzocaine | 12 | c | bc | ab | a | n.d. | H_3_= 34.181 (P < 0.001) |
| 1B vs 1E | MWRST | 10-20 | Benzocaine | 6 vs 12 | n.s. | n.s. | n.s. | n.s. | n.d. |  |
| 2A | KW (D) | 200-400 | Metacaine | 6 | c | bc | abc | a | ab | H_4_ = 32.289 (P < 0.001) |
| 2C | KW (T) | 200-400 | Metacaine | 12 | c | c | bc | ab | a | H_4_ = 42.599 (P < 0.001) |
| 2A vs 2C | MWRST | 200-400 | Metacaine | 6 vs 12 | T = 142.5 P = 0.005 | T = 136 P = 0.021 | T = 140 P = 0.009 | T = 155 P < 0.001 | n.s. |  |
| 2B | 1wA (HS) | 200-400 | Benzocaine | 6 | c | b | n.d. | a | n.d. | F_2,24_ = 18.831 (P < 0.001) |
| 2D | KW (T) | 200-400 | Benzocaine | 12 | b | b | n.d. | a | n.d. | H_2_= 20.729 (P < 0.001) |
| 2B vs 2D | MWRST | 200-400 | Benzocaine | 6 vs 12 | T = 134 P = 0.031 | T = 141 P = 0.007 | n.d. | T = 63 P = 0.006 | n.d. |  |
| 3A | KW (T) | 600-1300 | Metacaine | 6 | b | ab | a | n.d. | n.d. | H_2_= 6.356 (P = 0.042) |
| 3B | KW (T) | 600-1300 | Metacaine | 12 | b | ab | a | n.d. | n.d. | H_2_= 6.358 (P = 0.042) |
| 3A vs 3B | MWRST | 600-1300 | Metacaine | 6 vs 12 | n.s. | n.s. | n.s. | n.d. | n.d. |  |

**^1^**The post hoc tests are given in the parenthesis, D = Dunn’s method, T= Tukey test, HS= Holm-Sidak method

* To evaluate differences between temperatures, Mann-Whitney Rank Sum Test (MWRST) was performed

n.s. = not significant

n.d. = not determined (not included in the study)
