# Supplementary material for: Development of anaesthetic protocols for lumpfish (Cyclopterus lumpus L.): Effect of anaesthetic concentrations, sea water temperature and body weight
Source: PLoS One. 2017 Jul 5;12(7):e0179344. doi: 10.1371/journal.pone.0179344 (PMC5497946; doi:10.1371/journal.pone.0179344)
Supplement: S6 Table — (DOCX) [file pone.0179344.s006.docx]

**S6 Table. Statistical analyses to Figure 1-3 summarizing the effect of concentrations on time for normal respiration and effect of temperature.**

| **Corresponding Figure** | **Stat test^1*^** | **Fish Size (g)** | **Anaesthetic** | **Temp  (° C)** | **Induction time** | | | | | **Significance** |
| --- | --- | --- | --- | --- | --- | --- | --- | --- | --- | --- |
|  |  |  |  |  | **Anaesthetic concentration (mgL^-1^)** | | | | |  |
|  |  |  |  |  | **10** | **20** | **40** |  |  |  |
| 1C | KW (T) | 10-20 | Isoeugenol | 6 | b | b | a |  |  | H_2_ = 14.237 (P < 0.001) |
| 1F | KW (T) | 10-20 | Isoeugenol | 12 | - | - | - |  |  | n.s. |
| 1C vs 1F | MWRST | 10-20 | Isoeugenol | 6 vs 12 | n.s | n.s. | T = 136 P = 0.021 |  |  |  |
|  |  |  |  |  | **100** | **200** | **400** | **800** | **1600** |  |
| 1A | KW (D) | 10-20 | Metacaine | 6 | c | c | bc | ab | a | H_4_ = 41.794 (P < 0.001) |
| 1D | KW (T) | 10-20 | Metacaine | 12 | c | c | bc | ab | a | H_4_ = 43.863 (P < 0.001) |
| 1A vs 1D | MWRST | 10-20 | Metacaine | 6 vs 12 | T = 119.5 P = 0.018 | T = 149.5 P < 0.001 | T = 141.5 P = 0.006 | T = 142.5  P = 0.005 | T = 145 P = 0.003 |  |
| 1B | 1wA (HS) | 10-20 | Benzocaine | 6 | c | c | b | a | n.d. | F_3,39_ = 38.635 (P < 0.001) |
| 1E | KW (T) | 10-20 | Benzocaine | 12 | c | bc | ab | a | n.d. | H_3_ = 31.025 (P < 0.001) |
| 1B vs 1E | MWRST | 10-20 | Benzocaine | 6 vs 12 | n.s. | n.s. | n.s. | n.s. | n.d. |  |
| 2A | KW (D) | 200-400 | Metacaine | 6 | c | bc | abc | a | ab | H_4_ = 33.798 (P < 0.001) |
| 2C | KW (T) | 200-400 | Metacaine | 12 | c | bc | bc | ab | a | H_4_ = 36.704 (P < 0.001) |
| 2A vs 2C | MWRST | 200-400 | Metacaine | 6 vs 12 | T = 148 P = 0.001 | n.s | T = 141 P = 0.007 | T = 155 P < 0.001 | n.s. |  |
| 2B | KW (D) | 200-400 | Benzocaine | 6 | b | ab | n.d. | a | n.d. | H_2_ = 7.782 (P = 0.02) |
| 2D | KW (T) | 200-400 | Benzocaine | 12 | b | b | n.d. | a | n.d. | H_2_ = 19.582 (P < 0.001) |
| 2B vs 2D | MWRST | 200-400 | Benzocaine | 6 vs 12 | T = 141 P = 0.007 | T = 155 P < 0.001 | n.d. | T = 60 P = 0.017 | n.d. |  |
| 3A | 1wA (HS) | 600-1300 | Metacaine | 6 | b | b | a | n.d. | n.d. | F_2_ = 10.330 (P = 0.02) |
| 3B | KW (T) | 600-1300 | Metacaine | 12 | b | ab | a | n.d. | n.d. | H_2_ = 6.358 (P = 0.042) |
| 3A vs 3B | MWRST | 600-1300 | Metacaine | 6 vs 12 | n.s. | n.s. | n.s. | n.d. | n.d. |  |

**^1^**The post hoc tests are given in the parenthesis, D = Dunn’s method , T= Tukey test, HS= Holm-Sidak method

* for comparison between temperatures Mann-Whitney Rank Sum Test (MWRST) were used

n.s.= not significant

n.d. = not determined (not included in the study
